# An Overview of Molecular Mechanisms in Fabry Disease

**DOI:** 10.3390/biom12101460

**Published:** 2022-10-12

**Authors:** Federica Amodio, Martina Caiazza, Emanuele Monda, Marta Rubino, Laura Capodicasa, Flavia Chiosi, Vincenzo Simonelli, Francesca Dongiglio, Fabio Fimiani, Nicola Pepe, Cristina Chimenti, Paolo Calabrò, Giuseppe Limongelli

**Affiliations:** 1 Inherited and Rare Cardiovascular Diseases, Department of Translational Medical Sciences, University of Campania “Luigi Vanvitelli”, Monaldi Hospital, 80131 Naples, Italy; 2 Department of Nephrology, Monaldi Hospital, Via L. Bianchi, 80131 Naples, Italy; 3 Department of Ophthalmology, Monaldi Hospital, Via L. Bianchi, 80131 Naples, Italy; 4 Department of Neurology, Monaldi Hospital, Via L. Bianchi, 80131 Naples, Italy; 5 Molecular Genomics Lab., Chemical Biochemistry Unit, Monaldi Hospital, Via L. Bianchi, 80131 Naples, Italy; 6 Area ANMCO Malattie Rare—Dipartimento di Scienze Cliniche Internistiche Anestesiologiche e Cardiovascolari, Università La Sapienza, 00155 Rome, Italy; 7 Institute of Cardiovascular Sciences, University College of London and St. Bartholomew’s Hospital, London WC1E 6DD, UK

**Keywords:** Fabry disease, α-galactosidase A, biomarkers, mutations, *GLA* gene

## Abstract

Fabry disease (FD) (OMIM #301500) is a rare genetic lysosomal storage disorder (LSD). LSDs are characterized by inappropriate lipid accumulation in lysosomes due to specific enzyme deficiencies. In FD, the defective enzyme is α-galactosidase A (α-Gal A), which is due to a mutation in the *GLA* gene on the X chromosome. The enzyme deficiency leads to a continuous deposition of neutral glycosphingolipids (globotriaosylceramide) in the lysosomes of numerous tissues and organs, including endothelial cells, smooth muscle cells, corneal epithelial cells, renal glomeruli and tubules, cardiac muscle and ganglion cells of the nervous system. This condition leads to progressive organ failure and premature death. The increasing understanding of FD, and LSD in general, has led in recent years to the introduction of enzyme replacement therapy (ERT), which aims to slow, if not halt, the progression of the metabolic disorder. In this review, we provide an overview of the main features of FD, focusing on its molecular mechanism and the role of biomarkers.

## 1. Introduction

Fabry disease (FD) (OMIM #301500) is a rare disease with a prevalence of 1/80,000 live births [1]. The disease is a multisystem disorder characterized by a progressive lysosomal accumulation of neutral glycosphingolipids, in particular globotriaosylceramide (Gb3), in the tissues and organs of the whole body [2,3,4,5]. This accumulation is caused by partial (or total) deficiency of the enzyme α-galactosidase A (α-Gal A) [6], resulting from a mutation of the *GLA* gene present in the short arm of chromosome X (Xq22.1) [7]. Progressive accumulation of Gb3 is associated with a wide range of signs and symptoms of the disease, including kidney failure, cardiovascular dysfunction, neuropathy, stroke and dermatological manifestations in the form of angiokeratomas [2,3,8]. Age of onset and clinical presentation of FD largely depend on gender and degree of α-Gal A deficiency. Life expectancy is reduced by an average of 15 years in female patients [9] and 20 years in males [10]. The diagnostic suspicion is confirmed or ruled out by systematic screening of high-risk individuals with clinical features of FD, such as late-onset hypertrophic cardiomyopathy (HCM), cryptogenic stroke and end-stage renal disease [11,12,13,14,15,16].

## 2. Phenotypic Variability

The clinical manifestations of Fabry disease are slowly progressive, with variable onset, severity, age and course. The disease affects both females and males, but because it is an X-linked disease, males are more severely affected. There are two forms of FD in male patients: one with early onset and one with late onset. In the first case, a typical cluster of symptoms manifest in early childhood or early adolescence. In these patients, little or no enzymatic activity of α-galactosidase A is observed. Early symptoms include acute and chronic neuropathic pain, hypohidrosis, cutaneous angiokeratomas, gastrointestinal symptoms, lenticular and corneal opacities, microalbuminuria or proteinuria. With advancing age, these symptoms progress to renal failure, cardiomyopathy, cerebrovascular involvement and early death [17]. Acroparesthesias and pain may also occur, which can be triggered by heat and fever, but these symptoms are often misinterpreted and only occasionally lead to the correct diagnosis [18]. These manifestations occur in both boys and girls and can affect quality of life (QoL) [19]. Children with FD generally do not develop stroke, end-stage renal failure or heart failure, but there are early signs of cerebrovascular, renal and cardiac involvement [20]. The correct diagnosis is often delayed, although the first manifestations of FD occur in childhood [21,22]. Patients with a late-onset phenotype, whose symptoms appear between the third and sixth decades of life, have residual α-galactosidase A activity of up to 10% of normal [2]. This condition is able to “compensate” for an enzyme deficiency in which cardiac and/or renal symptoms appear in compliant adulthood and are often not recognized [23]. In both forms, the disease leads to progressive deterioration of renal status, cardiovascular involvement and cerebrovascular manifestations with age. These worsening symptoms, or a combination of them, may cause patients to die prematurely in their fourth or fifth decade of life [3]. Although death from complications associated with Fabry disease before adulthood is very rare, most affected males die by the end of their sixth decade of life if not adequately treated [9,10,24,25,26]. In the presence of the above-mentioned manifestations (e.g., multisystemic manifestation in classic form or unexplained left ventricular hypertrophy, stroke or chronic renal disease in later-onset form), FD should be considered in the differential diagnosis.

Penetrance in heterozygous females is 70% [27]. In contrast to most X-linked disorders, clinical symptoms in most heterozygous females are generally less severe and occur later than in affected hemizygous males [28,29]. However, in rare cases, females may also present with a range of severe manifestations similar to those seen in males [9,30]. The most common clinical manifestations in women include central nervous system impairment and cardiac disease, but they do not usually develop renal failure [31]. The lower severity and overall variability of the disease in women is partly due to the effects of X chromosome inactivation [9,10,25,31,32]. Current understanding of disease expression in women with X-linked disorders suggests that the severity of manifestations depends on the extent to which the normal X chromosome is inactivated. This wide clinical variability in both male and female patients with Fabry disease could be explained by genetic variability, by the numerous mutations described in the literature (more than 770) and by the still-unclear role attributed to intronic mutations and SNPs (single-nucleotide polymorphisms).

## 3. Molecular Mechanisms of Fabry Disease

FD is a lysosomal enzyme disease caused by a functional defect of the enzyme α-Gal A. The defect is due to mutations in the *GLA* gene located on the long arm of the X chromosome (Xq22.1) [6]. The coding portion consists of 1290 base pairs (bp) and is divided into seven exons (92–291 bp). There are several possible regulatory elements in the 5′-flanking region and an unmethylated CpG-rich island upstream of the initiation codon characteristic of a housekeeping gene [7,33,34]. The mRNA (1.45 kb) is unusual in its lack of a 3′-UTR sequence and encodes a protein, α-Gal A, of 429 amino acids, including the 31-amino-acid-long N-terminal signal peptide. Mutation of this gene causes an enzyme deficiency, responsible for the accumulation of two neutral glycosphingolipids, globotriaosylceramide (Gb3) and digalactosylceramide (GSL), in the lysosomes of tissues throughout the body, and particularly in those of the vascular endothelium. The result is progressive multisystemic, cellular and microvascular dysfunction. To date, more than 770 mutations in coding regions have been described in FD patients. However, there is no clear and unambiguous genotype–phenotype correlation [33,34]. In most patients with FD, “private mutations” can be observed (i.e., they occur in only one or a few families) [35,36,37].

**Figure 1:**
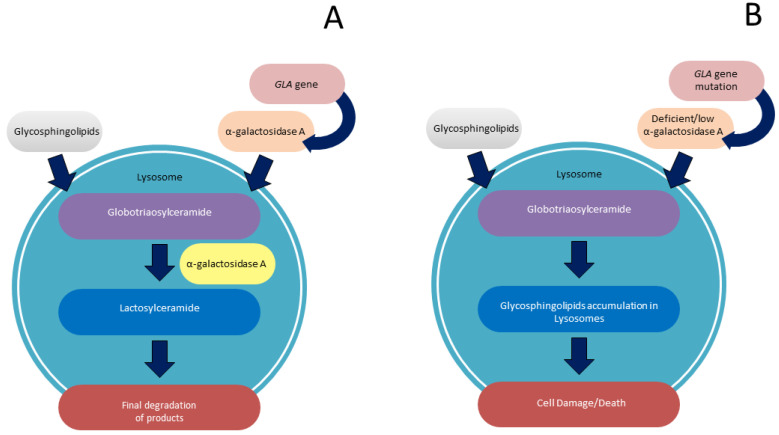
(**A**): In physiological condition, inside the lysosome, α-galactosidase A catalyses the hydrolysis of glycosphingolipids, with terminal α-galactose residues in the course of sphingolipid degradation; (**B**) in Fabry Disease, deficiency of *GLA* activity results in defects in degradation of glycosphingolipids, causing abnormal accumulation of enzyme substrate in the lysosome.

**Figure 2:**
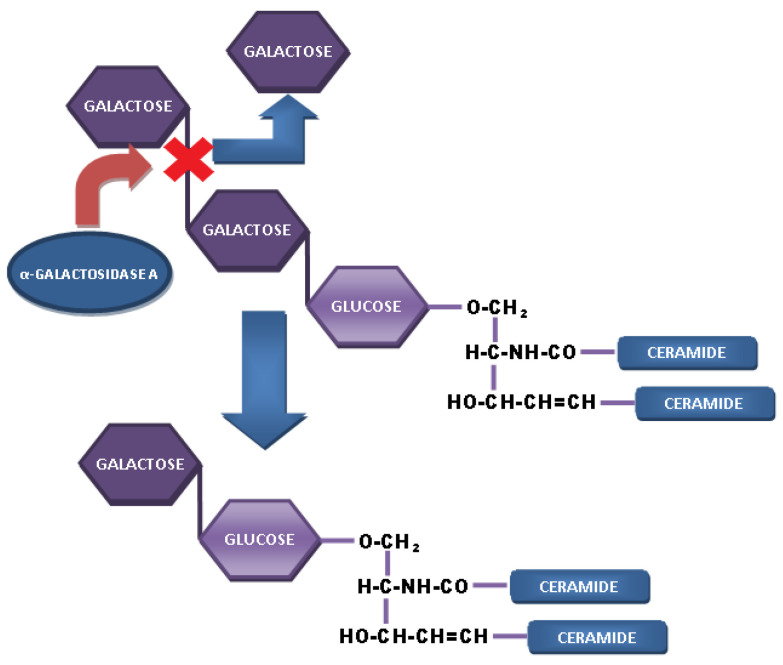
The enzyme α-galactosidase A cleaves off the third sugar residue. In Fabry disease, this enzyme is deficient, and therefore the substrate Gb3 accumulates in the lysosomes of almost all cell types.

Therefore, it is possible to observe different phenotypes even in one and the same family. In rare cases, male patients have been known to carry two different *GLA* mutations on the same allele, such as p.Glu66Gln and p.Arg112Cys or p.Leu89Arg and a 1 bp deletion in codon 303 [38,39]. In contrast, approximately 5% of patients have a pathogenic *GLA* mutation and the non-disease-associated variant p.Asp313Tyr. Mutations of the *GLA* gene include point mutations (missense: 55.9%; nonsense: 11.2%; at splice sites: 4.2%), “short-length” rearrangements (with about 60 nucleotides: 26.8%) and large rearrangements (involving one or more exons: 1.9%). The nonsense, missense and premature stop codons that lead to the absence or reduced activity of the enzyme α-Gal A are usually associated with the classic early-onset FD, characterized in males by the onset of symptoms in childhood, by multiorgan involvement and by the rapid progression of the disease, with clinical manifestations that often involve the heart, kidneys and central nervous system [40,41,42]. Early-onset FD is a serious condition that can have a significant impact on a person’s quality of life. While there is no cure for FD, early diagnosis and treatment can help to improve outcomes and slow the progression of the disease. These data were collected and recorded in the Fabry Outcome Survey (FOS). The FOS is a long-term surveillance study to describe the natural history of FD and the response to enzyme replacement therapy in a large cohort of European patients [2,43]. Therefore, large deletions or insertions (affecting up to ten nucleotides) are rare, as are complex mutations, while smaller deletions or insertions account for approximately 25% of all known disease alleles [33]. Many of these mutations are listed at Fabry-Database.org (http://fabry-database.org/, accessed on 11 October 2022). The study of the mutations has shown that there are no ‘hot spots’ of mutations, but that a high percentage of point mutations in exon 5 (3.15/10 bp) have been observed. This can be explained by the large size of the exonic portion (162 bp). Point mutations are also detectable in exon 6 (2.83/10 bp) and in exon 3 (2.53/10 bp) [44]. The enzyme α-Gal A is a glycoprotein synthesized as a precursor of 50 kDa, which is converted to a mature lysosomal form of 46 kDa via mannose-6-phosphate (M6P) after removal of a signal peptide during transport to the lysosome [34]. It is a homodimeric enzyme, and each monomer consists of two domains: one containing the active site and the other a β-sandwich of antiparallel β-strands at the C-terminus. Each monomer contains five disulphide bonds and four N-glycosylation sites. The active enzyme is a lysosomal acid hydrolase that requires saposin B to act on its natural substrates in vivo [45] and exhibits optimal activity towards natural and synthetic substrates at pH 3.8/4.6 [46] (Figure 1A). The identification of the three-dimensional structure of the protein using a crystallographic model has led to a better understanding of how the enzyme functions at the molecular level and to a better understanding of FD. Garman and Garboczi classified *GLA* missense mutations into three groups [47]. Mutations can result in a significant loss of metabolic activity of α-Gal A, although the protein may still retain some residual enzymatic activity [24] (Figure 1B). Glycosphingolipids such as Gb3 are components of the plasma membrane that are degraded in the lysosome. Their degradation requires the synergistic action of several hydrolysing enzymes, including α-Gal A (Figure 2). Deficiency of this hydrolase results in a progressive accumulation of the incompletely degraded Gb3 substrate in the cells. The debris is stored in multivesicular bodies or as intracytoplasmic masses and leads to cellular dysfunction or degeneration [48]. Mutations can therefore disrupt the active site of the enzyme by altering the residues that form it or that are essential for its correct three-dimensional structure. Alternatively, mutations may affect residues that are far from the active site, negatively affecting the folding and stability of the protein.

Finally, there are mutations that do not fit into any of the above categories (breaking of an important disulphide bond or elimination of an N-carbohydrate attachment site) [47]. In another study, mutations were identified that cause a premature stop codon, resulting in the creation of a truncated and nonfunctional protein, if not a complete absence of the gene product (functional null allele) [49].

## 4. The Role of Gb3 in FD

The cardiovascular involvement of FD is extremely important as it usually manifests as left ventricular hypertrophy (LVH), hypertrophic cardiomyopathy (HCM), myocardial fibrosis, heart failure and arrhythmias, which shorten the patient’s life span and lead to death if left untreated [37,40,41,50]. This is due to the accumulation of Gb3, which affects all types of cardiac cells and tissues (myocytes, endothelial and smooth muscle cells of intramyocardial vessels, endocardium, valve fibroblasts and conduction tissue) [51]. The main consequences include progressive LVH and diastolic production. Involvement of the intramural vessels leads to structural and functional changes, causing myocardial ischaemia [52]. As a result, there are progressive fibrosis and conduction abnormalities with the development of ventricular arrhythmias.

However, studies have shown that in addition to Gb3 accumulation, dysfunction also contributes to the development of Fabry cardiomyopathy. The results of several recent studies suggest that glycosphingolipids may play a role in the development of Fabry disease. These lipids are known to impair endocytosis and autophagy, triggering apoptosis [53] and interfering with mitochondrial energy production [54]. In addition, they can alter ion channel expression [55] and/or cell membrane transport, leading to electrical abnormalities in cardiomyocytes [56]. Furthermore, there is clinical and experimental evidence also support the role of glycosphingolipids in promoting inflammation through activation of Toll-like receptor-4 [17,57,58,59,60]. These findings suggest that glycosphingolipids may play an important role in the development of Fabry disease and that further research is warranted.

## 5. Inheritance

In the past, FD was classified as an X-linked recessive hereditary disease. However, women who carry the mutated gene may also show signs and symptoms of the disease. For this reason, FD is now described as an “X-linked transmission” disease, and the use of the term “carrier” for women carrying a disease-causing mutation may no longer be appropriate [61]. In fact, women may be affected as severely as hemizygous men due to the random inactivation of the normal X chromosome [62]. Men inherit the mutant *GLA* gene only from their mother (as it is located on the X chromosome) and are therefore always hemizygous for the Fabry gene. Consequently, males only pass on the mutated gene to their own daughters. Females are usually heterozygous for the mutations in *GLA* gene. While it is possible for a woman to have the mutated gene on both X chromosomes, the homozygous form is extremely rare [63]. A woman who is heterozygous for a mutation in *GLA* gene has a 50% chance of passing the disease to her offspring, regardless of sex. Due to lyonization (the random inactivation of one of the two X chromosomes), the activity of α-Gal A in affected women can range from low to normal [64]. Affected hemizygotes (males) have very low or undetectable enzymatic activity (except for those with the N215S mutation [34], who have higher residual activity in plasma and/or leukocytes). Therefore, heterozygotes cannot be reliably determined by enzyme analysis. Diagnosis must be made by sequencing, which allows for identification of the specific mutation in the *GLA* gene [63,65]. Over time, widely used enzyme assays have been developed for diagnosis [66], carrier detection [67] and prenatal identification of FD [68].

## 6. Diagnosis Algorithm

**Figure 3:**
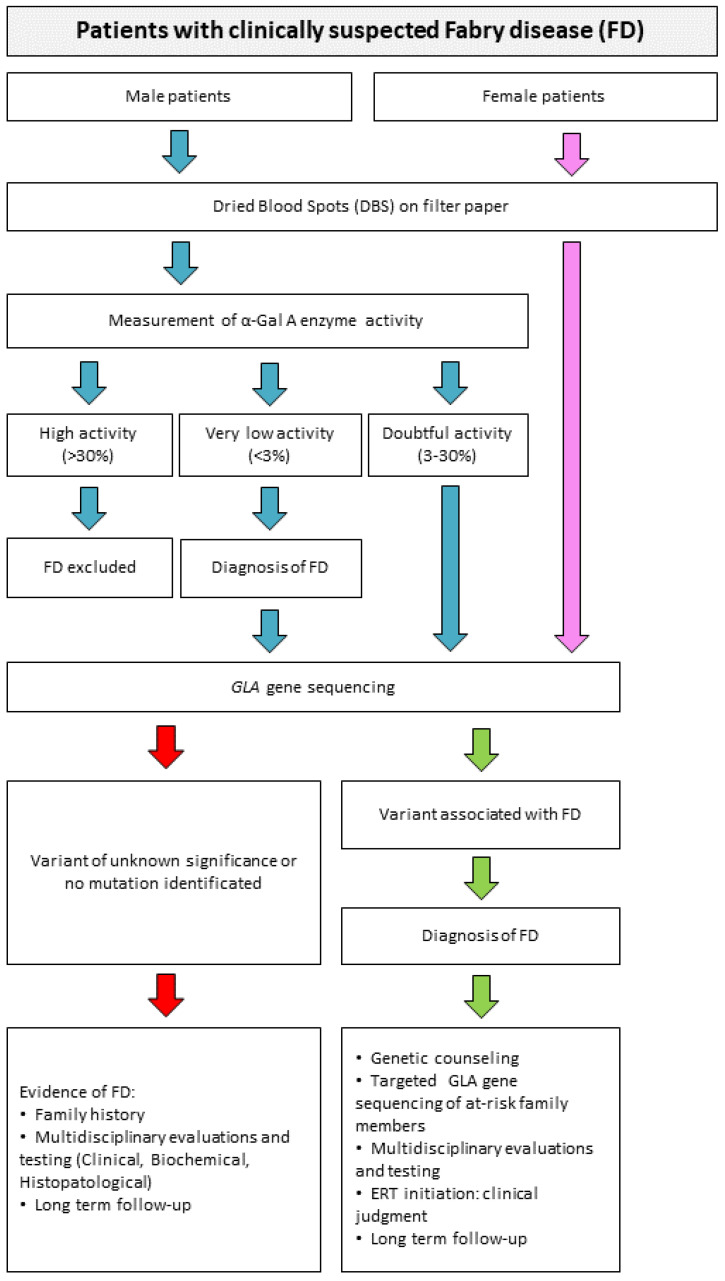
Algorithm for the diagnosis of Fabry disease. Starting from a diagnostic suspicion, it is possible to perform analyses that can confirm or reject the possible diagnosis of FD. Everything starts with DBS on filter paper, then the path changes in the case of male or female subjects. In male subjects, it is possible to perform a fairly rapid and exhaustive screening test of the dosage of α-Gal A enzyme activity. In case of a positive result (i.e., low enzymatic activity) or in case of doubt, it may be decided to perform sequencing of the 7 exons of the *GLA* gene to identify possible mutations causing the disease. In female subjects, where enzymatic activity may not be a diagnostic index, sequencing must be performed directly.

Often the diagnosis of FD is made late, when the damage caused by the disease is already irreversible [69]. Newborn screening and studies in high-risk patients can enable early diagnosis and thus the initiation of effective enzyme replacement therapy [70]. It is essential that awareness of this disease is improved to allow for early diagnosis and treatment. Early diagnosis and intervention before the onset of the disease will bring significant benefits to many patients and provide parents with the opportunity to receive genetic counselling. This variability is reflected in the wide range of specialists who diagnose the disease. As a result, many patients with FD go from one specialist to another and often receive an incorrect diagnosis. Diagnosis in male patients is mainly based on the typical signs and symptoms of the disease, while also assessing the activity of α-galactosidase A. The activity of α-GAL A can be easily measured in plasma [71,72], serum, urine and leukocytes [71,73] using the synthetic substrate 4-methylumbelliferil-α-d-galactopyranoside [49,71,74,75]. α-GAL A is also used to screen newborns with FD, as effective treatments are now available that can halt or delay clinical progression [70]. Screening is conducted using dried blood spots (DBS) on filter paper [76,77,78,79,80]. In case of positivity or doubtful result, molecular confirmation by the sequencing of the *GLA* gene is required (as in the diagnosis of women). Figure 3 shows the current algorithm used for the diagnosis of FD. DBS assay is now the most commonly used to investigate the activity of α-galactosidase A [79]. Compared to other assays, this method has several advantages, such as the high stability of the enzyme in the spots, the small amount of blood required and the rapidity and reliability of the analysis. Chamoles et al. [77] laid the foundation for the development of diagnostic assays using DBS for the detection of α-galactosidase activity. The assay is performed using portions of DBS together with a substrate, 4-methylumbelliferyl-α-d-galactopyranoside, and in the presence of a high concentration of N-acetylgalactosamine [49,71]. Fuller et al. [81] reported the immunoquantification of α-galactosidase A from DBS for the diagnosis of Fabry hemizygotes, making minor modifications to the previous protocol. New types of assays have emerged from these studies that can directly measure both the amount of lysosomal protein and the reaction rate in DBS. These assays are easily adapted to newborn screening [82]. These tests use fluorometric, radiometric, immunochemical and electrospray ionization tandem mass spectrometry assays (ESI-MS/MS). These types of tests identify only two-thirds of heterozygotes [83]. Meikle and colleagues have developed a method for newborn screening for FD using immunoquantification assays and tandem mass spectrometry [82,84]. Another method to evaluate the activity of α-GAL A is to examine the amount of the major accumulation product in different tissues. Gb3, a hallmark of FD, can indeed be quantified to establish the diagnosis of FD. Therefore, numerous methods have been developed to detect and measure Gb3, including thin-layer chromatography [85], gas–liquid chromatography [86], high-performance liquid chromatography [87] and an enzyme-linked immunosorbent assay using the B subunit of verotoxin [88].

Currently, gene sequencing is the method of choice for screening all female patients and for confirming the diagnosis in males with low α-galactosidase A activity for FD. Similar to other inherited diseases, genetic testing is mandatory in Fabry disease [89,90,91,92]. Many mutations in the *GLA* gene are capable of abolishing or significantly reducing α-galactosidase A activity. Today, targeted sequencing of the seven coding exons (including the promoter and adjacent and flanking intronic regions) of the *GLA* gene is the gold standard for molecular diagnosis of FD [36,93]. Advances in high-throughput NGS have enabled the use of genetic panels containing the *GLA* gene for screening high-risk patients, including people with HCM. This has resulted in the identification of many *GLA* variants of unknown significance (VUS) [94,95,96]. However, sequencing may in some cases fail to detect large deletions or duplications, leading to failure of the technique. Indeed, large gene rearrangements and cryptic splice site mutations in heterozygous females or affected males are not detected by targeted sequencing [97,98]. It should also be borne in mind that the *GLA* gene is rich in Alu repeat elements (approximately 1 Alu repeat per kb) [99] and that Alu–Alu recombination in heterozygotes for FD may be sequencing negative. In general, large genetic rearrangements can be investigated by Southern blot hybridization using a full-length cDNA probe or by PCR multiplex of the gene divided into four fragments. Single-base changes or small deletions and insertions can be detected by single-strand conformation polymorphism analysis [35], chemical cleavage of mismatches with fluorescence detection or denaturing high-performance liquid chromatography (DHPLC) [100]. Once the proband mutation is identified, a specific test is developed to screen family members. A specific mutation can be rapidly detected by DHPLC analysis of the amplified fragment containing the mutation, and subsequently confirmed by sequencing [100].

## 7. Biomarkers

The major diagnostic challenge of cardiovascular involvement in FD is that there is no single pathological phenotype. The significant overlap with other cardiovascular pathologies in terms of structural and functional alterations such as HCM and other storage pathologies involving the myocardium represents a complex of correct and early diagnosis. Some of the biomarkers normally used in clinical practice may be useful in the cardiovascular diagnosis process of Fabry patients. The dosage of cardiac troponins seems to be related to the presence of myocardial areas affected by necrosis and fibrosis [101]. Furthermore, the pathological processes of ventricular remodelling and deterioration of diastolic function are associated with an increase in the serum concentration of B-type natriuretic peptide (BNP or amino-terminal fragment of BNP). Concentrations of this marker correlate with disease severity and may be considered a prognostic marker [102].

The deacylated derivative of Gb3, globotriaosylsphingosine (Lyso-Gb3), was identified as a hallmark of FD [103]. Nowak et al. have shown that Lyso-Gb3 is, to date, the major metabolite of FD and a potential marker of disease progression. It has been associated with an increased risk of mortality. Lyso-Gb3 accumulation appears to worsen long-term clinical outcomes, but effective treatment should mitigate the cumulative toxic effects of Lyso-Gb3 [104].

In addition, recent studies consider the Lyso-Gb3 level as a possible diagnostic marker. Determination of plasma lyso-Gb3 levels should be considered for assessing disease severity in FD patients or in the diagnostic algorithm for patients with genetic GLA variants of unknown significance [42]. Therefore, Lyso-Gb3 has been suggested as a useful tool for predicting the pathogenicity of established VUS [105].

Thus, in patients with FD, there is reduced or undetectable α-Gal-A enzyme activity and progressive accumulation of glycosphingolipids, especially globotriaosylceramide (Gb3) and its deacylated form globotriaosylsphingosine (Lyso-Gb3), and gradual damage to cells throughout the body, including vascular endothelial cells, smooth muscle cells and cardiomyocytes [106].

For this reason, early diagnosis of FD represents a promising strategy to reduce organ damage, morbidity and premature mortality, which represents a clinical challenge, especially in patients with the nonclassic phenotype or who are asymptomatic. In this case, it is very important to have noninvasive biomarkers that allow for early detection and prediction of disease progression.

Lyso-Gb3 levels have been observed to be elevated in patients with genetic mutations that determine the classic phenotype, while they tend to be less elevated or normal in the “late-onset” variants or in benign mutations [41]. Furthermore, Lyso-Gb3 levels may provide useful information in patients with variants of unknown significance (VUS) in the *GLA* gene [107]. The potential prognostic role of Lyso-Gb3 dosage is controversial and continues to be hotly debated. However, recent data encourage it to be considered as a potentially useful marker for stratifying cardiovascular risk in patients with FD [104].

Recently, several alternatives have been proposed for monitoring FD patients. Several recent studies recommend miRNAs as a new class of biomarkers in FD, in particular mitochondrial miRNAs (mitomiRs). J. Gambardella et al. have demonstrated significant dysregulation of mitomiRs in FD patients. This dysregulation is probably an intimate mechanism of FD, independent of GB3. The concentration of mitomiRs could therefore be useful to monitor the progression of FD and highlight potential new therapeutic targets [108].

## 8. Therapies for FD

Recent advances in molecular biology and genetic engineering have enabled the development of therapies for FD [40]. Enzyme replacement therapy (ERT) has been successfully used to treat FD [70]. Prior to the introduction of ERT, treatment was essentially palliative and aimed to relieve symptoms. ERT studies in animal models of Fabry disease and other LSDs showed that intravenously infused lysosomal enzymes are rapidly taken up by the liver, spleen and other peripheral tissues, but do not normally reach the brain parenchyma [109]. Some of the problems associated with the use of ERT are the reduced efficacy of therapy at the CNS level, the immune response to the replacement protein, the use of lifelong therapy and the high cost of treatment [48,76].

**Figure 4:**
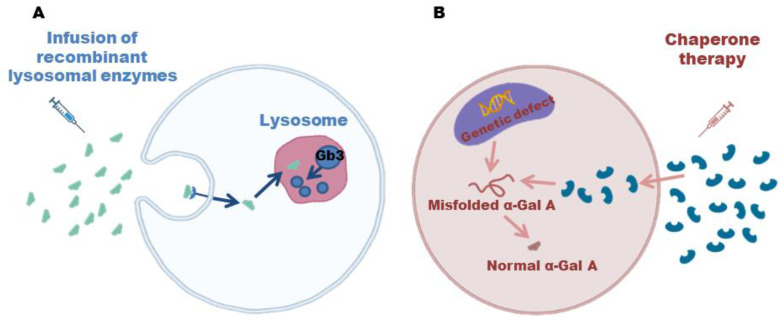
(**A**): The accumulation of Gb3 in tissues is a major contributor to the progression of the disease. However, thanks to intravenous infusion of recombinant lysosomal enzymes, this accumulation can be prevented, slowing down the disease; (**B**) enzyme enhancement therapy is based on oral use of chaperones that stabilize defective α-Gal A, promote proper folding and transport to lysosomes where accumulated substrates can be degraded.

ERT was developed thanks to the possibility of cloning the *GLA* gene and structural analysis of the enzyme, which allowed for the production of recombinant human α-galactosidase A (Figure 4A) [48,70].

This type of therapy allows for the reduction of endothelial inclusions of Gb3 in the heart [48,88,110,111] and in the vascular endothelium [111], as it provides pathologically absent or functionally altered α-Gal by biweekly intravenous administration of recombinant human α-Gal or β-Gal A [112]. This replacement therapy has resulted in excellent cardiovascular outcomes with consistent reductions in cardiac manifestations [48,70]. It is indicated in all symptomatic patients with classic disease, including children, at the first signs of organ involvement. Long-term follow-up studies have shown that ERT halts or slows disease progression and reduces the burden of clinical events [113]. However, potentially harmful anaphylactic reactions associated with antiagalsidase antibodies have also been reported [114]. ERT has had a profound impact on the natural history of FD and has significantly improved the quality of life of patients by effectively treating neuropathic pain, gastrointestinal manifestations and intolerance to heat and physical exercise. This is a significant breakthrough in the treatment of this debilitating disease [41,42,112]. Long-term follow-up studies are significant in terms of the potential for ERT to delay the progression of cardiac disease and reduce the cardiovascular event rate. The evidence suggests that early treatment with ERT may be effective in preventing LVH, and that mild LVH may even regress in some patients. However, the data are limited in terms of the effect of ERT on advanced cardiac FD, and it is not clear if the treatment has any effect on myocardial fibrosis or LVH progression [41,42,112].

Another ERT strategy for FD is enzyme enhancement therapy, which is based on the oral use of chaperones, iminosugars that exploit the residual activity of the endogenous polypeptide (Figure 4B) [115]. The binding of the pharmacological chaperone to the active site of 𝛼-Gal A stabilizes the defective enzymes and promotes their correct folding and transport to the lysosomes, where the enzyme can degrade the accumulated substrates [116].

In both cases, the heart responds less well to therapy when the disease is advanced, especially in patients with extensive fibrosis [48,88,110,115]. Some patients develop progressive structural heart disease with treatment-resistant complications, particularly if ERT is initiated at advanced stages of disease [116,117]. In these patients, the benefit of ERT may be attenuated. Consequently, cardiovascular complications are now the leading cause of FD-related mortality [118].

Recent studies have shown the beneficial effects of ERT, with the observed benefit being greater the earlier the therapy is started—at a young age, or in any case, before irreversible organ damage [113]. Therefore, it is important to emphasize the concept of early diagnosis and newborn screening in order to start treatment as early as possible in order to achieve greater benefit for the patient [116].

## 9. Conclusions

FD is a rare X-linked disorder that can occur before adolescence or later in life. In this disease, the α-Gal A enzyme may have decreased or absent activity. Affected males are always hemizygous and can be diagnosed by testing α-Gal A activity in plasma and/or leukocytes. The absence or reduced activity of the enzyme must be confirmed by the presence of mutations in the *GLA* gene. Heterozygotes (females) cannot be reliably diagnosed by enzyme testing, so genetic analysis is required. In some individuals, α-Gal A activity is below the normal reference range due to the presence of a pseudodeficiency allele. This condition can be confirmed by mutational analysis. Recent advances in our understanding of the complexity of cardiac FD have significantly improved diagnostic and therapeutic approaches. Although ERT has significantly changed the natural history of FD, cardiac involvement remains a key prognostic determinant. Cardiac manifestations benefit from early ERT, but clinical effects are limited in more advanced cases. New studies provide important insights into the limitations of enzyme replacement therapy (ERT) as a treatment for Fabry disease. This highlights the need for further research into the efficacy of ERT in the treatment of Fabry disease to ensure that patients receive the best possible care. A deeper understanding of secondary pathogenic pathways, particularly myocardial inflammation, may influence future therapeutic strategies. Although new disease-specific therapies appear promising, diagnostic delay and timely initiation of current treatments remain critical concerns for many patients with FD. Therefore, collaboration between FD specialists and cardiologists remains essential to identify patients before the onset of cardiac involvement, to enable them to gain maximum benefit from current and future therapeutic approaches.

## Data Availability

Not applicable.
